# Supplementary material for: Pre-clinical efficacy of a C4BP hexameric IgG Fc fusion protein against Neisseria gonorrhoeae
Source: PLoS One. 2026 Aug 25;21(8):e0350782. doi: 10.1371/journal.pone.0350782 (PMC13505937; doi:10.1371/journal.pone.0350782)

S1 Fig. Schematic of the C4BP-Hexa IgG Fc fusion protein and its characterization by SDS-PAGE and SEC-HPLC. (A) Schematic representation of C4BP-Hexa IgG Fc. (B) Representative protein gel of C4BP-Hexa IgG Fc with or without dithiothreitol (DTT) reducing reagent. (C) SEC-HPLC of C4BP-Hexa IgG Fc from *Nicotiana benthamiana* which was purified using protein A chromatography followed by Capto Core 400.

A

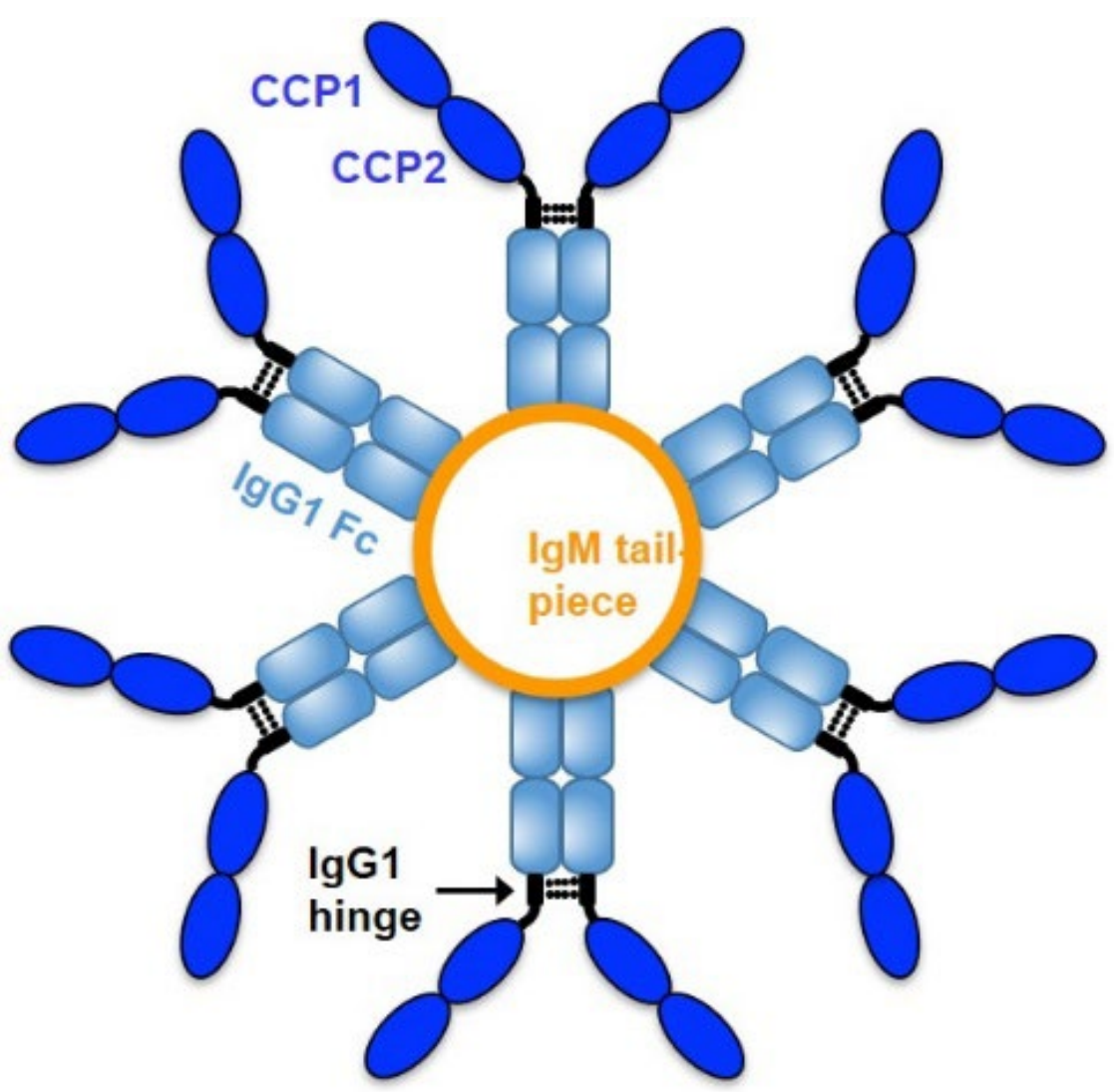

B

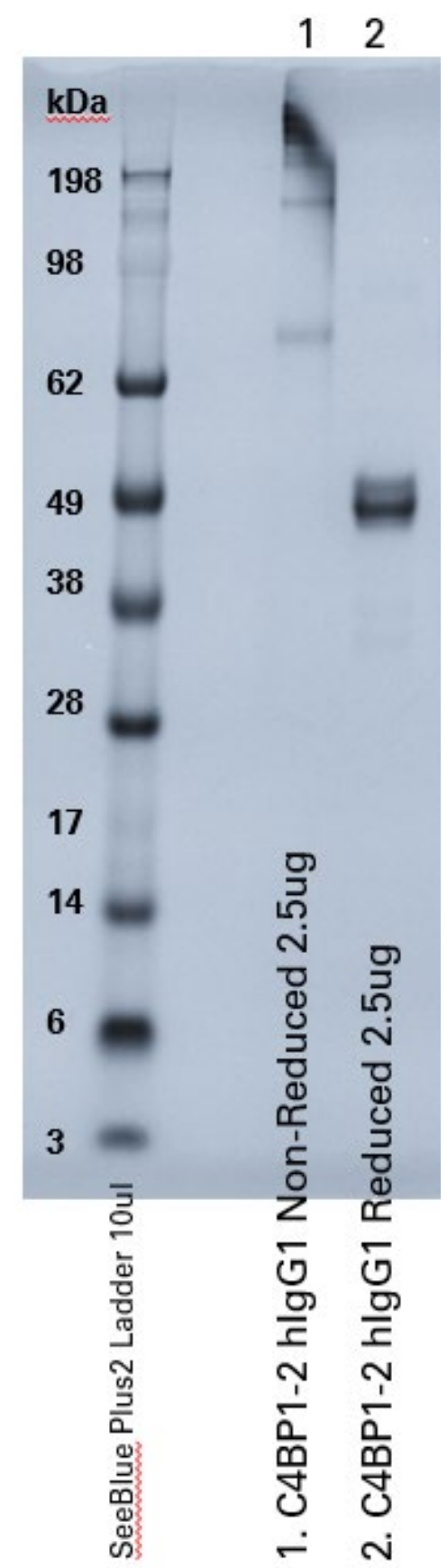

C

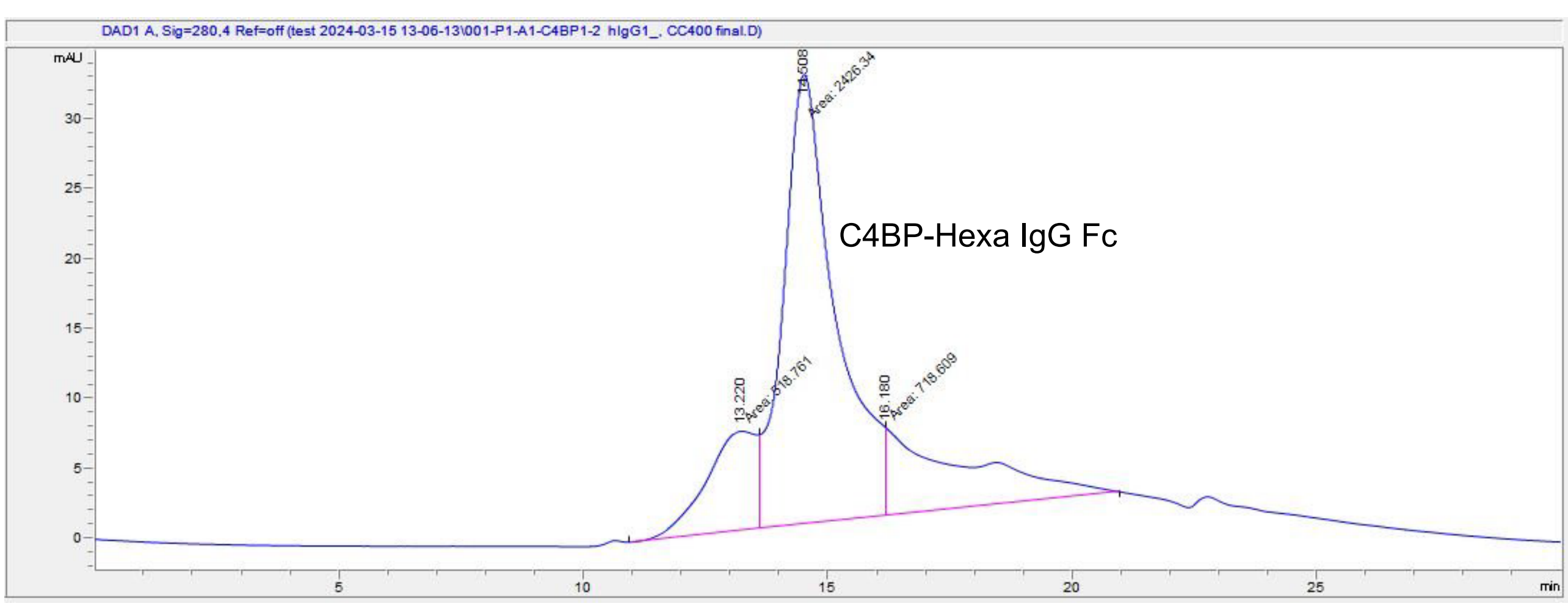

Supplement: Supplementary file 1 [file pone.0350782.s001.pdf]
